# Maladaptive coping among military-connected adolescents: Examining combined risk using QCA

**DOI:** 10.3389/fpsyg.2022.948474

**Published:** 2022-12-19

**Authors:** Tamika D. Gilreath, Francisco A. Montiel Ishino, Kathrine S. Sullivan, Titilayo A. Okoror

**Affiliations:** ^1^Transdisciplinary Center for Health Equity Research, Texas A&M University, College Station, TX, United States; ^2^National Institute on Minority Health and Health Disparities (NIH), Bethesda, MD, United States; ^3^Silver School of Social Work, New York University, New York, NY, United States; ^4^Department of Africana Studies, Binghamton University, Binghamton, NY, United States

**Keywords:** military-connected adolescents, maladaptive coping, social support, mental health, risk behavior

## Abstract

**Introduction:**

Military-connected students in public schools face a unique set of stressors that may impact their wellbeing and academic functioning.

**Methods:**

Twenty-four youth in the 7th to 12th grades who had an active-duty parent (mother or father) serving in the U.S. Armed Forces were interviewed. Participants completed a qualitative interview while actively completing a Life History Calendar (LHC) to mark deployment and family military service milestones and discuss how they impacted the youth respondent. This study used Qualitative Comparative Analysis (QCA) to explore the interplay and combination of specific stressors related to relocation and deployment experiences among adolescents, and to determine key factors associated with maladaptive outcomes.

**Results:**

The results of the QCA analysis identified bullying experiences and negative experiences with other military-connected youth as conditions that are associated with maladaptive coping.

**Discussion:**

Chronic and acute stressors in adolescence are established risk factors for mental, emotional, and behavioral problems in the short and long-term including suicidality, substance use and abuse, and substance use disorders. Through qualitative inquiry we were able to identify specific contextual details related to maladaptive coping that can be used to further refine areas of focus for research, prevention, and interventions for military-connected adolescents.

## Introduction

In the United States, there are nearly 1 million school-aged children with a parent in the military (National Academies of Sciences, 2019), and close to 1.2 million when those with a sibling who is serving are included (DePedro et al., 2011). The vast majority of United States-based military-connected students from both active duty and National Guard and Reserve families attend public schools. An additional 71,000 students attend 164 schools located in the United States and around the word that are accredited by the Department of Defense (DoD) and run by the Department of Defense Education Activity (DoDEA; DePedro et al., 2011; National Academies of Sciences, 2019). Military-connected students in public schools face a unique set of stressors that may impact their wellbeing and academic functioning. On average, military families move every 2–3 years (Park, 2011), and students from these families may change schools up to nine times in their lives (Esqueda et al., 2012). Though some youth appear to manage these transitions successfully (Weber and Weber, 2005), many highly mobile students find school transitions stressful (Mmari et al., 2010) and feel disconnected from school experiences (Capp and Sullivan, 2020).

Further, more than 2 million service members and their families have experienced an overseas deployment since 2001, with many families experiencing multiple deployments and reunifications during this period (National Academies of Sciences, 2019). Though some appear to weather deployment separations successfully, these experiences have been associated with changes in youth behavior, mental health, and academic performance (Huebner and Mancini, 2005; Chandra et al., 2010; Engel et al., 2010; Lester et al., 2011). The collective impact of exposure to stressors associated with wartime military life may explain increased rates of adverse outcomes including depressive symptoms, suicidality, substance use, and victimization observed among military-connected public school students (Reed et al., 2011; Millegan et al., 2013; Sullivan et al., 2015; Gilreath et al., 2016). Few studies have attempted to describe how youth experience deployment-related stressors in the context of psychosocial transitions (Flake et al., 2009; Chandra et al., 2010; Cozza, 2011; DePedro et al., 2011; Lester et al., 2011; Gilreath et al., 2013).

Specifically, there is a dearth of empirical data on the impact of the unique interplay, or combined impact, of relocations and deployments on adolescent behavioral health (Chandra et al., 2010). Among military-connected youth, the importance of social connections and social support emerge as critical protective factors that reduce the likelihood of adverse outcomes (Mmari et al., 2010; Lucier-Greer et al., 2015; Gilreath, 2016; Conforte et al., 2017). However, the experience of relocation and school transition make accessing supportive social relationships among both military and nonmilitary peers more challenging (Bradshaw et al., 2010) and may increase military-connected students’ risk for bullying and victimization (Sullivan et al., 2015). In addition to social relationships, school transitions also potentially disrupt academic progress as school requirements may differ between states (Bradshaw et al., 2010). These changes are often compounded by stress associated with deployment separation, which can also adversely impact academic engagement and performance for military-connected adolescents (Engel et al., 2010; Card et al., 2011; Nicosia et al., 2017).

Though many elements of the deployment cycle may be challenging for military-connected youth (Meadows et al., 2016), coping with post-deployment mental health changes of a parent can be particularly troubling. Specifically, post-traumatic stress disorder (PTSD) among military parents, which includes symptoms of avoidance, arousal, and hypervigilance, has been associated with poor parent–child relationships and adverse youth outcomes (Sullivan et al., 2016; Creech and Misca, 2017). Thus, factoring in the stress of parental deployments, the stress of war, and multiple relocations of schools and neighborhoods, it becomes clear that military-connected children experience significant life stressors.

Several studies have found general associations between deployments and/or relocations and maladaptive coping behaviors among military-connected youth (Bello-Utu and DeSocio, 2015). However, it is unclear what components of military stressors are most salient to maladaptive coping behaviors specifically. Thus, there is a need to examine mechanisms by which military-connected stressors increase maladaptive coping. Maladaptive coping behaviors, such as, poor academic performance, substance use, bullying/victimization have been found to be associated with deleterious health and social consequences (Catalano and Hawkins, 1996; Bello-Utu and DeSocio, 2015).

The present study uses Qualitative Comparative Analysis (QCA) to explore the interplay between, and outcomes associated with, specific stressors related to relocation and deployment experiences among adolescent public school students, and to determine key factors associated with maladaptive outcomes. Configurational methodologies like QCA are critical to understand the cumulative effects of stressors unique to military-connected youths’ maladaptive coping. In understanding the configurational pathways, whether singular or combinatorial, to maladaptive coping we can better design mental health measures for this unique group to develop targeted prevention and interventions programs.

## Materials and methods

### Participant sample

Participants were recruited from middle and high schools near a military installation in Southern California between October 2016 and March 2018. The present study includes 24 interviews from youth in the 7–12th grades who had an active duty parent (mother or father) serving in the United States Armed Forces. Fifty-eight percent (n = 14/24) of the sample were female with an average age of 14 (R = 12–17). Active duty affiliations were primarily with the US Marine Corps (87.5%; n = 21/24) while the remainder were from the US Navy (see Table 1).

**Table 1:** Sample demographics.

	Frequency/Count
**Race/Ethnicity**	
Asian	2
Black American	5
Latino/Hispanic	3
Mixed race/ethnicity	4
Native American/American Indian	1
White	9
**Sex**	
Female	14
Male	10
**Armed Forces Branch of Service**	
United States Marine Corps	21
United States Navy	3

Interviewers met with individual students, one-on-one, after school hours on school property. Parents provided written consent, and minor participants provided assent. Interviews lasted between one and one and a half hours. Participants completed a brief demographic questionnaire and then participated in the qualitative interview while actively completing a Life History Calendar (LHC) to mark deployment and family military service milestones and discuss how they impacted the youth respondent. Institutional Review Board approval was sought and received from the Texas A&M University Human Subjects Research Review Board.

### Interview protocol

Individual interviews were conducted using a semi-structured protocol guided by LHC approach (Freedman et al., 1988; Caspi et al., 1996). This approach utilizes a calendar as a visual aid to increase participants’ recall of salient events as well as their thoughts and feelings regarding these experiences (Freedman et al., 1988; Caspi et al., 1996). The calendar employed in this study was created as a laminated, poster-size Excel spreadsheet allowing participants to record their responses directly on the calendar as they interacted with the interviewers. Calendars were organized with years across the top (beginning with the current year and counting backwards to 2002 – depending on the student’s age at the time of interview) and relevant military salient domains (e.g., deployments, relocations) in the left-most column.

In addition to the calendar, interviews were guided by a semi-structured interview protocol that included probing questions about each domain. These questions were not intended to be prescriptive, but rather to provide direction to the interview process. Questions expanded upon domains included in the calendar and included such prompts as “Can you tell me about the experience(s) of learning your loved one was being deployed overseas? What did you understand your loved one would be doing while they were away?” All interviews were audio recorded. Upon completion of the interview, pictures were taken of the LHC as the participant had completed it.

### Qualitative data analysis and coding for selection of outcome and conditions

The interviews were digitally recorded, transcribed verbatim, and then manually coded using a grounded theory approach as delineated (Glaser and Strauss, 1967). This approach was used for the initial analysis of each interview, and then to analyze the content (Glaser and Strauss, 1967). No preformed hypotheses were used in the initial analysis. Open coding was used on each interview over several iterative stages. Each interview was first coded to identify concepts, followed by the identification and examination of themes. The qualitative coding team (TO and FAMI) agreed on the coding schema to reliably code for themes and achieve inter-rater reliability (IRR). IRR was established using Cohen’s kappa, which yielded a value of 0.81 after coding four interviews. NVivo (QSR International, Version 11) qualitative data analysis software was used to facilitate the coding process, and allowed the coding team to collaboratively work on coding the interview transcripts. Once themes were assessed, the following categories were identified as possible conditions to the outcome of maladaptive coping behaviors based on a review of existing literature: (1) military-connected youth that have been bullied by other youths; (2) military-connected youth’s experiences with enlisted parents living with PTSD; (3) military-connected youth’s negative experiences with other military-connected youth; and (4) academic problems.

### Operationalization of conditions and the outcome

Using a grounded theory approach, we were able to create a final list of outcomes and conditions that are detailed below with examples from the interviews in parentheses. Outcomes and conditions were created through calibration of codes from interviews, and were treated as binary data, i.e., based on conditional propositions where the conditions are either true/present [1] or false/absent [0]. We used calibration—the process of assigning the condition—for the crisp-set QCA (csQCA) outcomes and conditions. The qualitative coding team (TO and FAMI) calibrated the presence or absence of the given outcomes and conditions. All outcomes and conditions were based solely on the military-connected youth context and experience.

Maladaptive outcomes were identified by examining the situation and context, and then determining whether the reaction/coping strategy presents the opportunity for long-term improvement or serves as a temporary outlet with high potential for negative consequences (Zeidner and Saklofske, 1996). Youth in the present study discussed self-injury and improper management of anger/stress (“Oh, for me, I initially tried to make it a healthy way of coping, so I’d look it up online, and I found this method of using hair ties and flicking them against your wrist. And for the most part, that helped, and then that led me to do-I guess worse ways to cope with how I was feeling, and that eventually led to self-harm, and that eventually led to therapy. I’ve definitely had instances where that’s happened”…“Yeah, just like you take your anger out in a way that you should not-hit stuff, throw things, just normal ways of expressing anger in the wrong way. That can be bad. Or just like yelling at someone and hurting someone’s feelings, even though they did absolutely nothing wrong to you. They were just like being mean, or just nasty, just for no reason at all”).

Several potential explanatory conditions were identified in the interviews with youth. Youth reported being bullied at school (“I’ve never been bullied-bullied, I would say, but I have had experiences where people have either made - it was Missouri, I would say, where people would make fun of me, but like never like physical bullying, more like verbally bullying”), shared their experiences of having a parent living with PTSD (“But I can also kind of see how it’s changed him, so-I’m a follower, I’m not a leader, so I walk behind people a lot, because I feel most comfortable that way, but with my dad’s PTSD, no one can walk behind him because he’ll feel super unsafe”), and several participants noted how difficult it can be to interact with other military-connected youth (“People I know military I really cannot, like, connect that good with them, because I’m usually used to connecting with people that is not in the military, because we never - this is my first time living on base”). Finally, these military-connected youth often reported academic problems due to relocation (“Well, in […] they taught - I would say, they taught differently, like different things than they did here. So I would say, I was behind because I did not know most of the stuff that they were teaching”). Relocations, whether nationally or internationally, have consequences due to differences in schooling systems. Most youth reported having difficulties when introduced into a new school, while others found that relocation occurred during an enlisted parent’s deployment.

### Analytic plan

Qualitative comparative analysis is a mixed methods approach that allows the combination of qualitative and quantitative analytic tools first developed by Ragin (1987). Once outcomes and conditions were calibrated and assigned, we used csQCA to examine complex sets of binary data. Boolean algebra was used to identify patterns from the conditions. Cases, or the respondents/military-connected youth, are treated as configurations of conditions to an outcome. We would then test whether given combinations of conditions were in a subset or superset relationship to the outcome of interest, i.e., whether the condition is (not) Sufficient and/or (not) Necessary to produce an outcome. Sufficiency is when conditions co-occur with the outcome and Necessity is when conditions are always present when the outcome is present but do not guarantee the outcome. Of note is that solutions vary in complexity level. The use of Boolean minimization can provide the most parsimonious model describing the patterns of multiple conditions in the binary data to our given outcome. Boolean minimization also provides the simplest pathways to the outcome that demonstrates the highest consistency and coverage scores.

In the current study, maladaptive coping was investigated as the dichotomous outcome/condition. For our purpose, cases were military-connected youth and were similar, satisfying the conditions of being sufficiently parallel and necessarily comparable. The cases shared enough background characteristics, such as age and schooling, to be considered as constants in the analysis aside from being military-connected. We gained sufficient in-depth insight into different cases to be able to capture the complexity of cases and produce some level of generalization (Rihoux and Lobe, 2009).

Marx and Dusa (2011) describe nine steps needed for csQCA. The following are the steps that were undertaken to complete our analysis: (1) select a desired outcome to be examined (i.e., maladaptive coping in military-connected youth); (2) define the research population and select cases with enough variation on the outcome to be examined; (3) select and list conditions that may contribute to the outcome; (4) define each binary condition and outcome for the purposes of calibration using Direct Method () for transparency; (5) display a data matrix for each coded condition by case; (6) specify an explanatory model and resolve contradictions in the matrix; (7) create a truth table by transforming the matrix; (8) generate the most parsimonious explanation from the model; and (9) interpret the explanatory models (i.e., models that explain both the presence and absence of the outcome selected). We added an additional step to test the robustness of the resulting explanatory model with the outcome present. All analytic procedures for csQCA were performed on R Package QCA (Version 3.3; Dușa, 2018).

In csQCA all cases are either true or false (i.e., 1 or 0, respectively). The value of 1 became the inclusion set into full membership; conversely, the value of 0 became the exclusion set to full non-membership. The crossover point was set to 0.5 (Marx and Dusa, 2011), where values over 0.5 became part of the inclusion set, and values at or below 0.5 became part of the exclusion set. Thus, the raw qualitative data underwent a numerical transformation. Boolean methods are used to identify patterns of consistency, or how consistent a condition is present in the presence of an outcome, especially when examining small to medium-N purposive samples. To test the generalizability or validity of the observations, we assessed robustness using the retention() procedure in R Package QCA (Version 3.3). The procedure is a simulation based on the combinatorial method developed by Thiem et al. (2016) to calculate the retention. In addition to the retention procedure, we used the baQCA() procedure in R Package braQCA (Version 1.0.0.1) to test the robustness of the QCA solution to randomness through a bootstrap assessment (Ben Gibson and Vann, 2016).

## Results

Table 2 presents the truth table used for the QCA. Four participants had maladaptive coping behaviors and 17 did not. Three cases were intermediary, at 0.5 or below and were placed into the exclusion set. The outcome of maladaptive coping [MALCOPE] was observed in military-connected youths that have in combination: (1) been bullied [YESBBULL], had negative experiences with other military-connected youths [NEGMCY], and had academic problems [ACADPROB]; or (2) had been bullied [YESBBULL], did not have an enlisted military family member living with PTSD [mfmptsd], had not had negative experiences with other military-connected youths [negmcy], and had not had academic problems [acaprob]; or (3) had not been bullied [yesbbull], had an enlisted family member living with PTSD [MFMPTSD], had not had negative experiences with other military-connected youths [negmcy], and had academic problems [ACADPROB].

**Table 2:** QCA truth table.

	Explanatory conditions				Outcome		
	YESBBULL	MFMPTSD	NEGMCY	ACAPROB	MALCOPE	*N*	Incl.
1	0	0	0	0	0	10	0.2
2	0	0	0	1	0	2	0
3	0	0	1	0	0	2	0
4	0	1	0	0	0	1	0
5	0	1	0	1	1	1	1
6	1	0	0	0	1	1	1
7	1	0	0	1	0	3	0.3
8	1	0	1	1	1	1	1
9	1	1	0	0	0	2	0.5
10	1	1	1	1	1	1	1

YESBBULL, Military-connected youth that have been bullied by other youths; MFMPTSD, Military-connected youth’s experiences with enlisted parents living with PTSD; NEGMCY, Military-connected youth’s negative experiences with other military-connected youth; ACAPROB, academic problems; MALCOPE, maladaptive coping behavior; Incl., sufficiency inclusion score.

### Minimized reduced configurational expressions


YESBBULL∗NEGMCY→MALCOPE

In rewriting the equations described above and resolving all contradictions, the common configuration of conditions to maladaptive coping in military-connected youth is having been bullied [YESBBULL] and having had negative experiences with other military-connected youths [NEGMCY].

### Robustness

The robustness or retention probabilities for the csQCA solution, under various perturbation scenarios, are 0.817 and 0.857 with respect to corruption and deletion simulations. These values indicate that if the same study were to be conducted, 82 and 86% of the solutions would remain the same if participants were to shift conditions or drop out, respectively. Similarly, under a bootstrap assessment running 5,000 simulations, there is an 88.8% probability that the solution remains [5% CI: 0.8808; 95% CI: 0.8952].

## Discussion

Research has found that military-connected youth may be at higher risk than nonmilitary connected youth for adverse and maladaptive behavioral health outcomes (Reed et al., 2011; Gilreath et al., 2013, 2016; Sullivan et al., 2015; Nicosia et al., 2017). However, a limitation of the prior literature has been the lack of an in-depth examination of concurrent potential mechanisms leading to negative outcomes (Chandra et al., 2010). Correlates of negative outcomes have largely been limited to demographic characteristics, number of deployments, relocations, and military connection status (Reed et al., 2011; Gilreath et al., 2013, 2016; Sullivan et al., 2015). Survey responses provide only limited insight into types of maladaptive coping and underlying experiences that may negatively impact military-connected youth. The results of the QCA analysis identified bullying experiences and negative experiences with other military-connected youth as conditions that are associated with maladaptive coping. The present study builds on the prior literature by identifying and examining correlates of maladaptive coping through a more descriptive lens.

As noted previously, school transitions make accessing supportive social relationships among both military and nonmilitary peers more challenging (Bradshaw et al., 2010), and there is research to indicate that military-connected youth may be at increased risk for bullying, victimization, and discrimination in schools (Atuel et al., 2014; Sullivan et al., 2015). This is consistent with other studies that have identified social support as key in decreasing risk for negative behavioral health outcomes among military-connected adolescents (Gilreath, 2016; Conforte et al., 2017). Surprisingly, parental PTSD did not emerge as a salient factor in maladaptive coping (Sullivan et al., 2016; Creech and Misca, 2017). Research does however suggest that social support can ameliorate the effects of PTSD (Creech and Misca, 2017), and that this may be the case in the current sample.

Interestingly, of those interviewed who expressed negative experiences with other military-connected youth, the majority did indicate that these were associated with differences in interactions that occurred on-base compared to off-base. For example, one respondent indicated “People I know military I really cannot, like, connect that good with them…this is my first time living on base” and another stated “I felt like - I tried really hard being a part of the military, like the programs they had on base? Like the teen center we used to have on base?... I stopped involving myself with stuff like that.” Given the importance of peer group membership in adolescence, negative interactions with members of the same social group may be associated with increased likelihood for maladaptive outcomes (Newman et al., 2007).

The present study utilized qualitative interviews as a tool to gather detailed information about military-specific conditions of youth attending public schools. The use of QCA facilitates the summarization of detailed qualitative interview data into statistically robust equations/estimates of associations. QCA provided a possible explanatory model of the causal conditions that military-connected youth experience, and identified likely pathways to maladaptive coping strategies. However, this study does have limitations.

### Limitations

A calibration issue that must be addressed is the meaning of 0, or full exclusion of a case out of a set. The challenge to contend with in csQCA is what does the 0 actually mean? Does it mean a true absence or a non-mention? For our cases, zeros may be due to interviewer differences. Although all interviewers were trained in implementing the LHC survey, four interviewers were ultimately assigned to interview 24 participants (K1 = 4; P2 = 4; P3 = 9; G4 = 7). As each interviewer introduced their own style, a true absence to non-mention cannot be fully triangulated. Further, as these interviews were conducted in a single school district that serves one United States Marine Corps (USMC) base, the results may not be applicable to youth whose parents serve in other branches of the military or in other locations. Additionally, it is noteworthy that given the sample size and qualitative nature of the data collection, this study was not able to include results that are more detailed by age or gender *via* QCA. Other qualitative studies with military-connected youth, however, have identified similar concerns by military-connected youth with similar sample size (Houston et al., 2009; Thomas et al., 2021).

Chronic and acute stressors in adolescence are established risk factors for mental, emotional, and behavioral problems in the short and long-term including suicidality, substance use and abuse, and substance use disorders (Sinha, 2008). The extant literature indicates that the majority of daily stressors that adolescents will face revolve around social relationships (Seiffge-Krenke, 2004). Thus, war and transition-related relationship stress may put the adolescent children of service members, at high risk for engaging in maladaptive coping responses which may predispose them to a variety of adverse health and social outcomes throughout adolescence and into adulthood (Jessor, 1991; Newcomb et al., 1997; Irwin et al., 2002; Johnston et al., 2006; Eaton et al., 2010). Understanding the salient experiences that increase the likelihood of maladaptive coping and adverse outcomes continues to be a primary goal for military families research. Through qualitative inquiry, we were able to identify specific contextual details related to maladaptive coping that can be used to further refine areas of focus for research, prevention, and interventions for military-connected adolescents. Specifically, further assessment of gender differences in maladaptive coping might be useful for tailoring/targeting school supports. Further, there are likely varied manifestations of maladaptive coping between early (11–14) and late adolescents (15–17). There is also some evidence that there might be differential experiences depending on branch of affiliation for military-connected youth (Bello-Utu and DeSocio, 2015). Future research should include larger and more diverse samples to elucidate these nuanced mechanisms.

## Data availability statement

The datasets presented in this article are not readily available because data include qualitative interview recordings and are not feasible for sharing. Requests to access the datasets should be directed to TG, tgilreath@tamu.edu.

## Ethics statement

The studies involving human participants were reviewed and approved by Texas A&M University Institutional Review Board. Written informed consent to participate in this study was provided by the participants’ legal guardian/next of kin.

## Author contributions

TG designed and conducted the study and drafted manuscript. KS, TO, and FAMI conducted analyses, collaborated on drafts, and completed overall review of the manuscript. All authors contributed to the article and approved the submitted version.

## Funding

This work was fully supported by the Eunice Kennedy Shriver National Institute of Child Health and Human Development under Grant 1R21HD085149–01.

## Conflict of interest

The authors declare that the research was conducted in the absence of any commercial or financial relationships that could be construed as a potential conflict of interest.

## Publisher’s note

All claims expressed in this article are solely those of the authors and do not necessarily represent those of their affiliated organizations, or those of the publisher, the editors and the reviewers. Any product that may be evaluated in this article, or claim that may be made by its manufacturer, is not guaranteed or endorsed by the publisher.
